# Supplementary material for: Repression of LSD1 potentiates homologous recombination-proficient ovarian cancer to PARP inhibitors through down-regulation of BRCA1/2 and RAD51
Source: Nat Commun. 2023 Nov 16;14:7430. doi: 10.1038/s41467-023-42850-x (PMC10654398; doi:10.1038/s41467-023-42850-x)
Supplement: Supplementary file 1 — Supplementary Information [file 41467_2023_42850_MOESM1_ESM.pdf]

Repression of LSD1 potentiates homologous recombination-proficient ovarian cancer to PARP inhibitors through down-regulation of BRCA1/2 and RAD51

Lei Tao[1*], Yue Zhou[1*], Xiangyu Pan[1], Yuan Luo[1], Jiahao Qiu[1,2], Xia Zhou[1], Zhiqian Chen[3], Yan Li[4], Lian Xu[5], Yang Zhou[1], Zeping Zuo[1,6], Chunqi Liu[1], Liang Wang[1], Xiaocong Liu[1], Xinyu Tian[1], Na Su[7,8], Zhengnan Yang[1], Yu Zhang[9], Kun Gou[1], Na Sang[1], Huan Liu[2,7], Jiao Zou[1], Yuzhou Xiao[1], Xi Zhong[7], Jing Xu[1], Xinyu Yang[7], Kai Xiao[1], Yanyang Liu[1], Shengyong Yang[1], Yong Peng[1], Junhong Han[1], Xiaobo Cen[2] and Yinglan Zhao[1†].

[1]Department of Biotherapy, Cancer Center and State Key Laboratory of Biotherapy, West China Hospital, Sichuan University, Chengdu 610041, China.

[2]National Chengdu Center for Safety Evaluation of Drugs, State Key Laboratory of Biotherapy, West China Hospital, Sichuan University, Chengdu 610041, China.

[3]State Key Laboratory of Natural and Biomimetic Drugs, School of Pharmaceutical Sciences, Peking University, Beijing 100191, China.

[4]Department of Pharmacology, Shanxi Medical University, Taiyuan 030001, China.

[5]Department of Pathology, Key Laboratory of Birth Defects and Related Diseases of Women and Children of Ministry of Education, West China Second University Hospital, Sichuan University, Chengdu 610041, China.

[6]Laboratory of Anesthesiology & Critical Care Medicine, Department of Anesthesiology, West China Hospital, Sichuan University, Chengdu 610041, China.

[7]Department of Pharmacology, Key Laboratory of Drug Targeting and Drug Delivery System of the Education Ministry, Sichuan Engineering Laboratory for Plant-Sourced Drug and Sichuan Research Center for Drug Precision Industrial Technology, West China School of Pharmacy, Sichuan University, Chengdu 610041, China.

[8]Department of Pharmacy, West China Hospital, Sichuan University, Chengdu 610041, China.

[9]School of Medicine, Tibet University, Lhasa 850000, China.

†Corresponding author. Email: zhaoyinglan@scu.edu.cn

*These authors contributed equally to this work.

This PDF file includes:

Supplementary Fig. 1 to 11

Supplementary Table 1 to 2

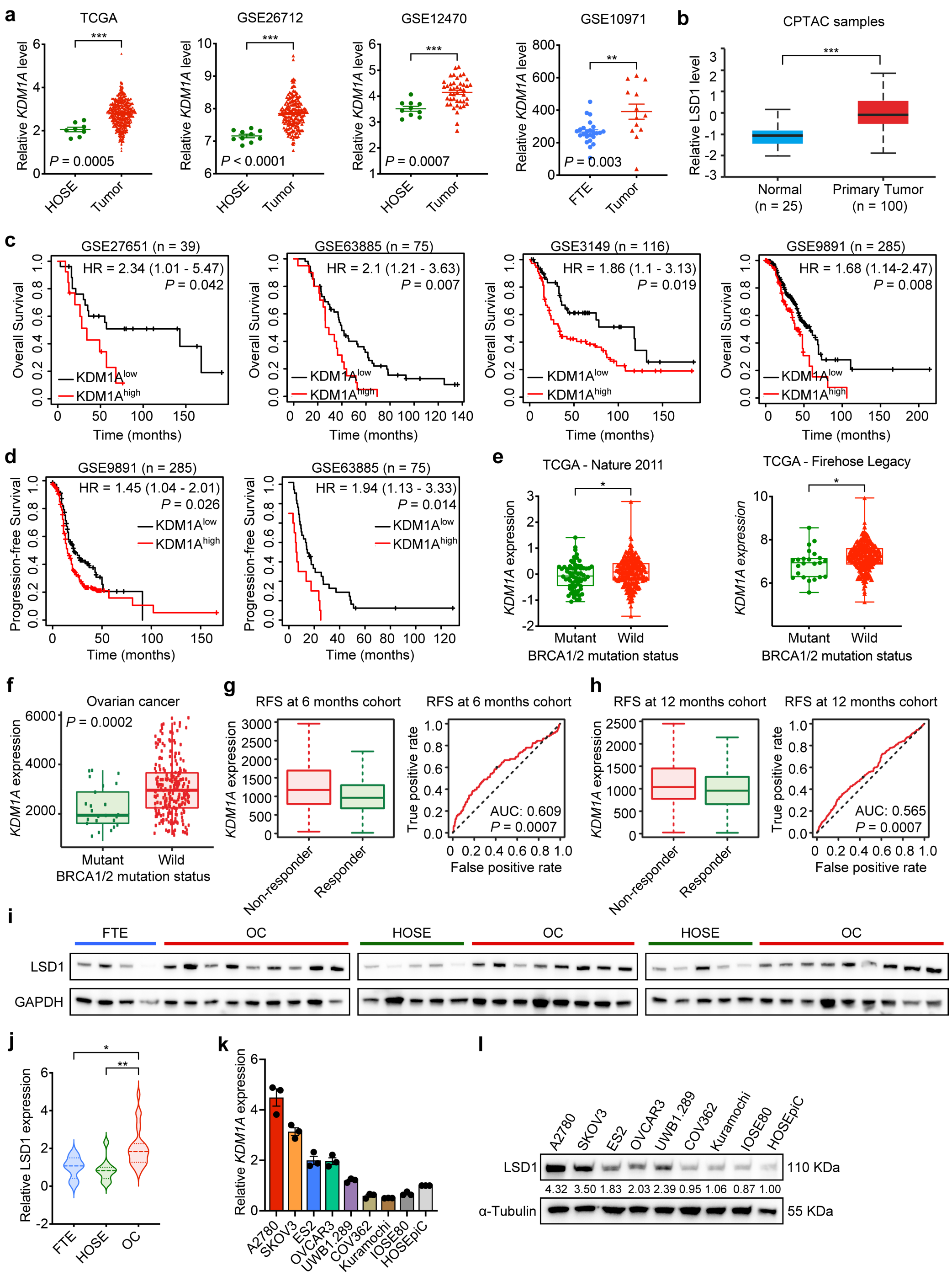

Supplementary Fig. 1 | Overexpression of LSD1 is observed in human OC tissues and correlated with poor survival of patients.

a, *KDM1A* expression in OC and normal tissues using TCGA, GSE26712, GSE12470 and GSE10971 dataset. Data represent mean ± SEM (unpaired two-tailed Student's *t* test).

b, Boxplot showing relative protein expression of LSD1 between normal and OC samples in Clinical Proteomic Tumor Analysis Consortium (CPTAC) samples using UALCAN web-portal (http://ualcan.path.uab.edu).

c, Kaplan-Meier plot depicting overall survival of OC patients with tumors expressing high (red) or low (black) levels of *KDM1A* using the online database (http://www.kmplot.com/) (log-rank test).

d, Kaplan-Meier plot depicting progression-free survival of OC patients with tumors expressing high (red) or low (black) levels of KDM1A using the online database (http://www.kmplot.com/) (log-rank test).

e, **f**, The boxplots showing *KDM1A* expression correlated with BRCA1/2 status using cBioPortal (**e**) and muTarget (**f**) platforms.

g, **h**, The boxplots and ROC curves of *KDM1A* gene expression levels and platinum response using the RFS at 6 (**g**) and 12 (**h**) months cohort. RFS, relapse-free survival. ROC, receiver operating characteristic.

i, Western blot analysis showing LSD1 level in the indicated human FTE, HOSE, and OC tissues. GAPDH was used as the loading control.

j, Quantification of immunoblot presented in (**i**) normalized to loading control by densitometry. Data represent mean ± SEM (unpaired two-tailed Student's *t* test).

k, LSD1 mRNA expression of the indicated human cell lines as determined by RT-qPCR analysis. Expression levels in each cell line were normalized to β-actin mRNA and expressed as fold change relative to HOSEpiC cells. Data represent mean ± SEM (n = 3 biologically independent experiments).

l, Western blot analysis showing LSD1 level in the indicated human cell lines. GAPDH was used as the loading control. Numbers below western blot panels represent relative quantification of the respective bands normalized to loading control by densitometry.

ns, not significant, $p > 0.05$; *$p < 0.05$; **$p < 0.01$; ***$p < 0.001$. Source data and exact *p* values are provided as the Source Data file.

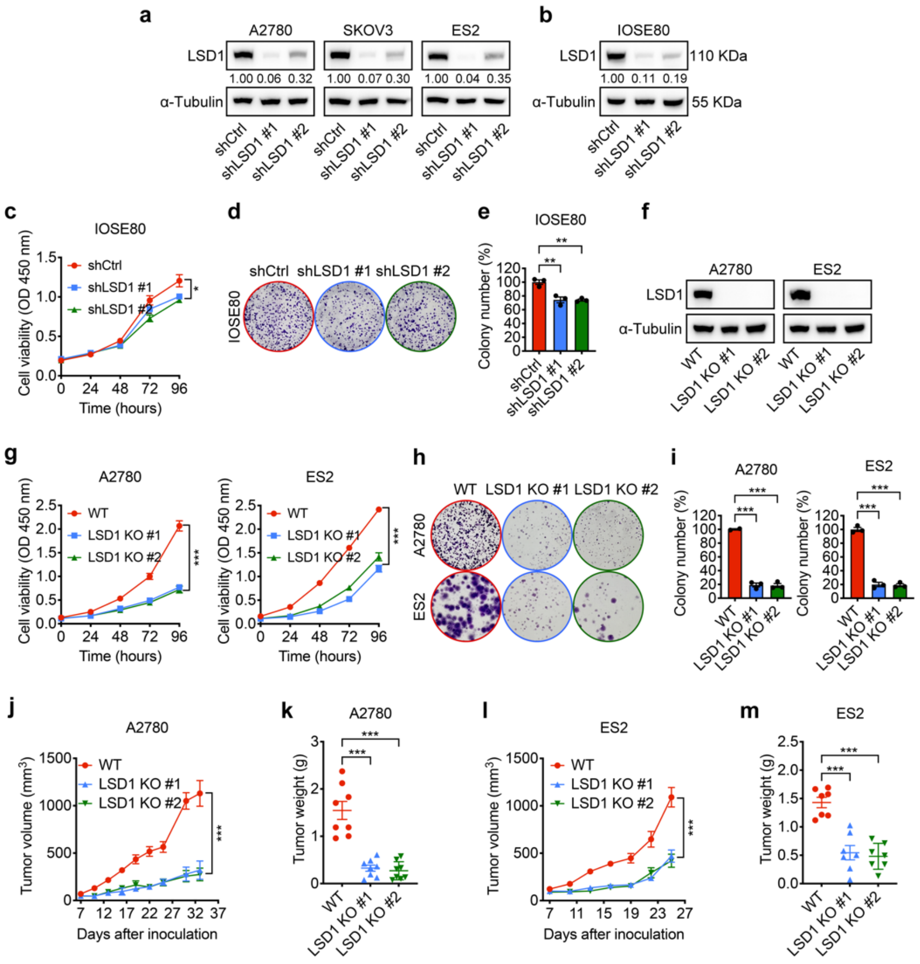

Supplementary Fig. 2 | LSD1 promotes OC progress.

a, b, Western blot analysis of LSD1 protein levels in A2780, SKOV3, ES2 and IOSE80 cells transduced with 2 different shRNAs targeting LSD1 (shLSD1 #1 and shLSD1 #2) or nontargeting control (shCtrl).

c, Cell growth of LSD1 knockdown IOSE80 cells (shLSD1 #1 and shLSD1 #2) detected by CCK8 assay compared with their control (shCtrl). Data represent mean ± SEM of three biologically independent experiments (two-way ANOVA).

d, **e**, Representative images (**d**) and quantification (**e**) of colony formation assay for IOSE80 cells with LSD1 knockdown (shLSD1 #1 and shLSD1 #2). Samples were normalized to nontargeting control shCtrl. Data represent mean ± SEM of three biologically independent experiments (unpaired two-tailed Student's *t* test).

f, Western blot of LSD1 protein in A2780 and ES2 cells before and after LSD1 knockout.

g, Cell growth of A2780 and ES2 cells before and after LSD1 knockout. Data represent mean

± SEM of three biologically independent experiments (two-way ANOVA).

h, i, Representative images (**h**) and quantification (**i**) of colony formation assay for A2780 and ES2 cells with LSD1 knockout. Data represent mean ± SEM of three biologically independent experiments (unpaired two-tailed Student's *t* test).

j-m, Tumor volume (**j, l**) and tumor weight (**k, m**) of A2780 and ES2 subcutaneous xenografts in nude mice before and after LSD1 knockout. Data represent mean ± SEM (n = 8 mice per group for A2780 xenograft models, n=7 mice per group for ES2 xenograft models; two-way ANOVA for panels **j, l**, and unpaired two-tailed Student's *t* test for panels **k, m**).

$p > 0.05$; *$p < 0.05$; **$p < 0.01$; ***$p < 0.001$. Source data and exact *p* values are provided as the Source Data file.

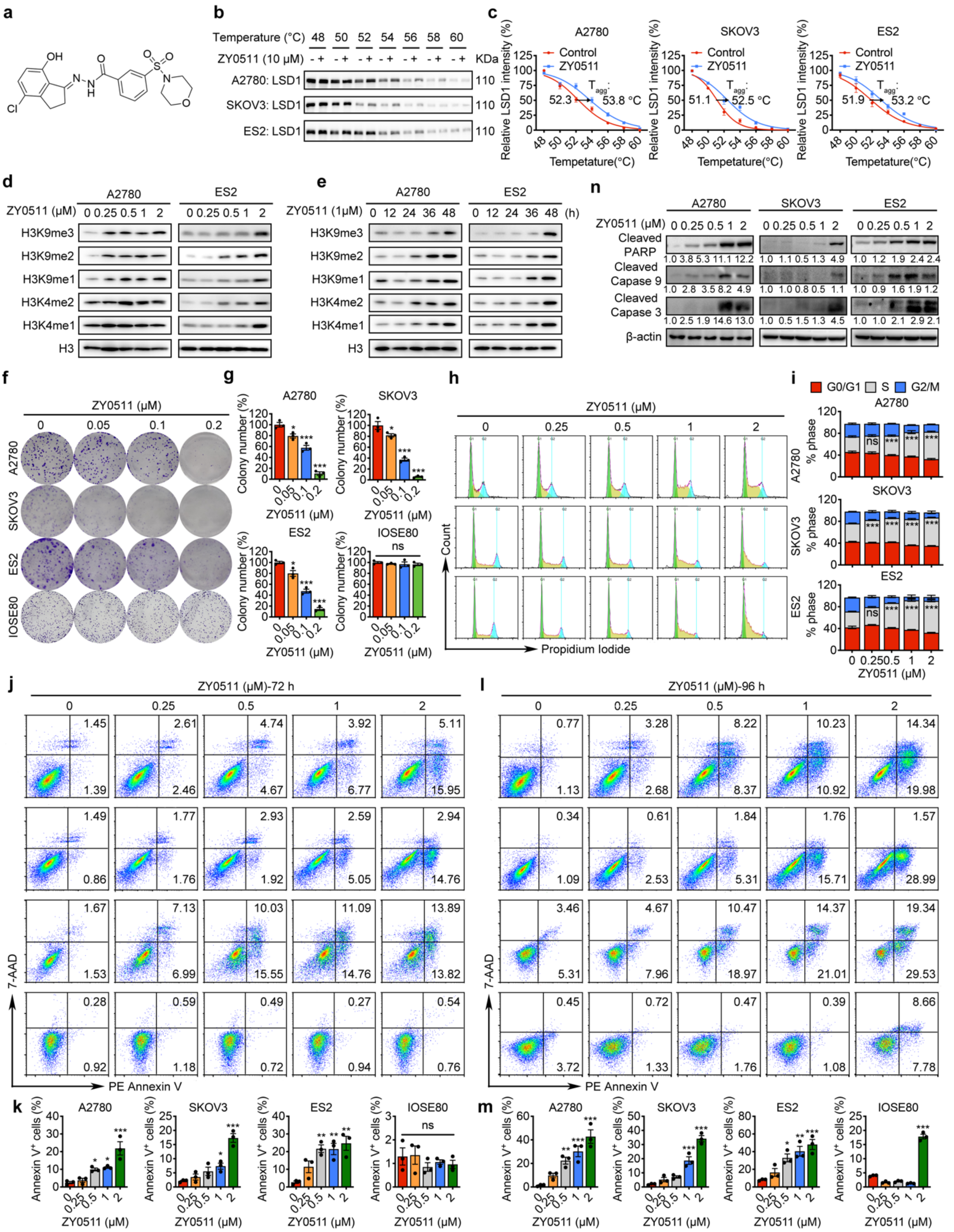

Supplementary Fig. 3 | ZY0511 is a potent and selective inhibitor of LSD1.

a, Chemical structure of ZY0511.

b, **c**, Representative (**b**) and quantification (**c**) of western blot images of CETSA in intact cells. The solid lines represent the best fits of the data to the saturation binding curve model

within the GraphPad Prism software. Data represent mean ± SEM (n = 3 biologically independent experiments). Abbreviations: CETSA, cellular thermal shift assay; T_{agg}, aggregation temperature.

d, e, Western blot analysis of indicated proteins in A2780 and ES2 cells treated with the indicated concentrations of ZY0511 for 48 h (**d**) or treated with 1 μM ZY0511 for the indicated time periods (**e**). Histone H3 was used as the loading control.

f, g, Representative images (**f**) and quantification (**g**) of the colony formation assay for indicated cells after ZY0511 treatment. Data represent mean ± SEM normalized to untreated cells (n = 3 biologically independent experiments; unpaired two-tailed Student's *t* test).

h, **i**, Representative images (**h**) and quantification (**i**) of cell cycle analysis after ZY0511 treatment for 24 h. Data represent mean ± SEM (n = 3 biologically independent experiments; unpaired two-tailed Student's *t* test).

j, **k**, Representative images (**j**) and quantification (**k**) of cell apoptosis analysis after ZY0511 treatment for 72 h. Data represent mean ± SEM (n = 3 biologically independent experiments; unpaired two-tailed Student's *t* test).

l, **m**, Representative images (**l**) and quantification (**m**) of cell apoptosis analysis after ZY0511 treatment for 96 h. Data represent mean ± SEM (n = 3 biologically independent experiments; unpaired two-tailed Student's *t* test).

n, Western blot analysis of indicated proteins in A2780, SKOV3 and ES2 cells treated with the indicated dose of ZY0511 for 48 h. β-actin was used as the loading control. Numbers below western blot panels represent relative quantification of the respective bands normalized to loading control by densitometry.

ns, not significant, $p > 0.05$; *$p < 0.05$; **$p < 0.01$; ***$p < 0.001$. Source data and exact *p* values are provided as the Source Data file.

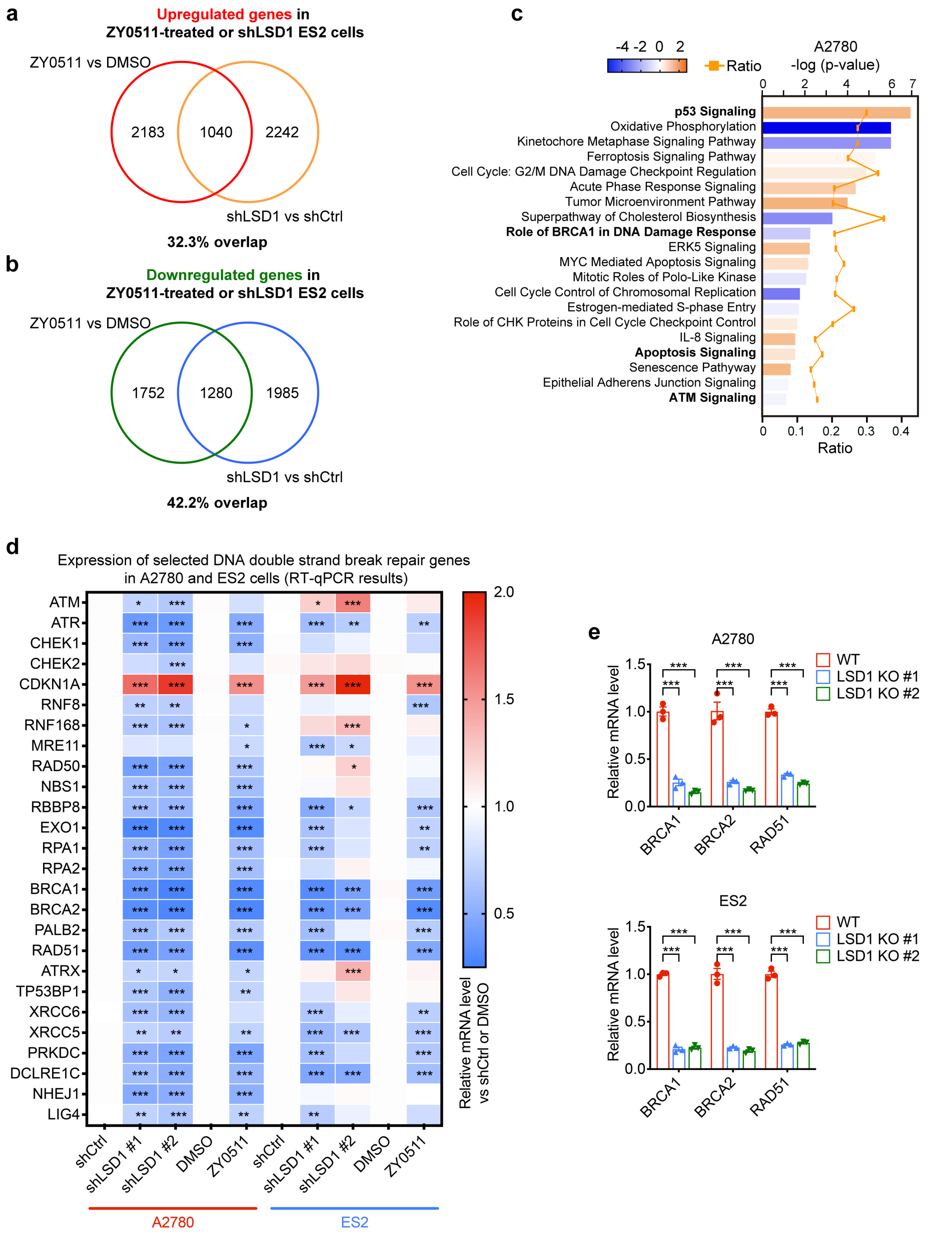

Supplementary Fig. 4 | Effect of LSD1 inhibition on gene expression.

a, **b**, Venn diagram showing the overlap of the up- or down-regulated genes in LSD1 knockdown and LSD1i (ZY0511)-treated ES2 cells.

c, Ingenuity Pathway Analysis of the representative twenty significantly regulated pathways of LSD1i (ZY0511)-treated versus untreated A2780 cells. Upregulated pathways are presented in orange and downregulated pathways are in blue. *p* values generated by right-tailed Fisher's exact test.

d, Heatmap of RT-qPCR analysis of indicated gene expression at 36 h with LSD1i (ZY0511)-treated versus untreated control or LSD1 knockdown (shLSD1 #1 and shSLD1 #2) versus nontargeting control (shCtrl) in A2780 and ES2 cells. GAPDH was used as the loading

control (n = 3 biologically independent experiments; unpaired two-tailed Student's *t* test).

e, RT-qPCR analysis of indicated gene expression after LSD1 knockout in A2780 and ES2 cells. GAPDH was used as the loading control (unpaired two-tailed Student's *t* test).

$p > 0.05$; $*p < 0.05$; $**p < 0.01$; $***p < 0.001$. Source data and exact *p* values are provided as the Source Data file.

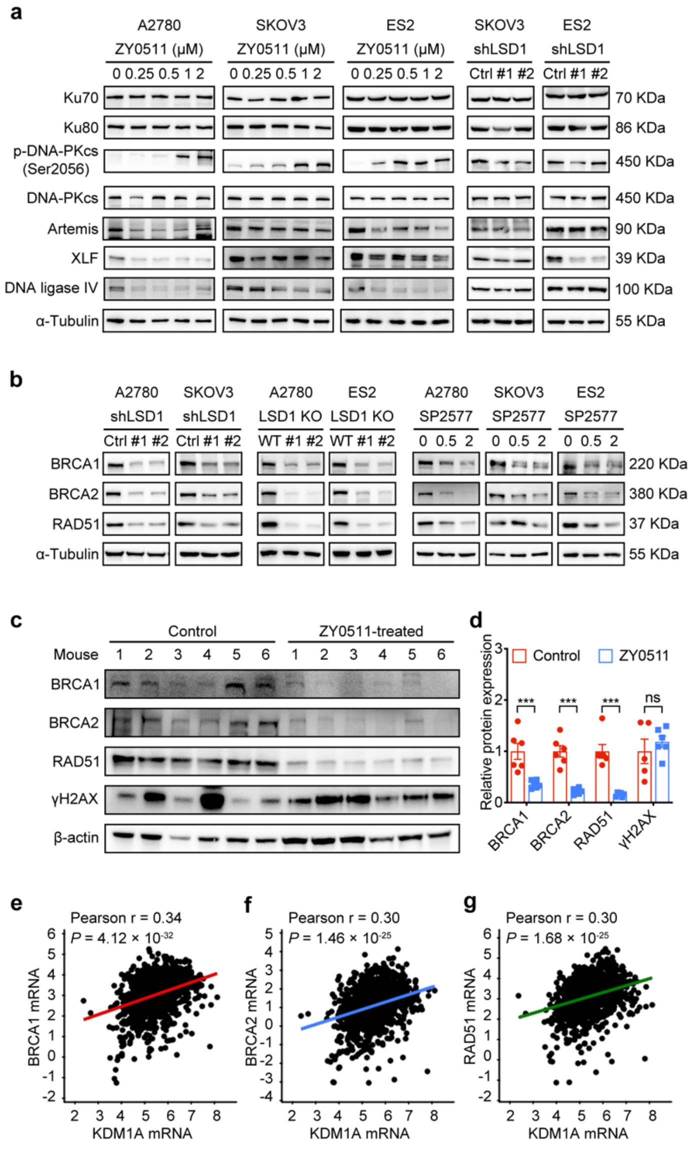

Supplementary Fig. 5 | Effect of LSD1 inhibition on HR and NHEJ protein expression.

a, **b**, Western blot analysis of indicated proteins in A2780, SKOV3 and ES2 cells treated with the indicated dose of LSD1i (ZY0511 or SP2577) for 48 h, or treated with shRNAs targeting LSD1 (shLSD1 #1 and shLSD1 #2) and nontargeting control (shCtrl), or before and after LSD1 knockout. α-Tubulin was used as loading control.

c, Western blot analysis of indicated proteins in tumor tissues from SKOV3 subcutaneous xenografts treated with the indicated compound or vehicle at the end of the experiment. β-actin was used as loading control.

d, Quantification of experiment presented in (**c**) normalized against β-actin. Data represent mean ± SEM (n = 6 mice per group; unpaired two-tailed Student's *t* test).

e-g, Correlation between LSD1 and BRCA1 (**e**), BRCA2 (**f**) and RAD51 (**g**) mRNA expression

in Cancer Cell Line Encyclopedia (Broad, 2019) database.

ns, not significant, $p > 0.05$; $^{*}p < 0.05$; $^{**}p < 0.01$; $^{***}p < 0.001$. Source data and exact *p* values are provided as the Source Data file.

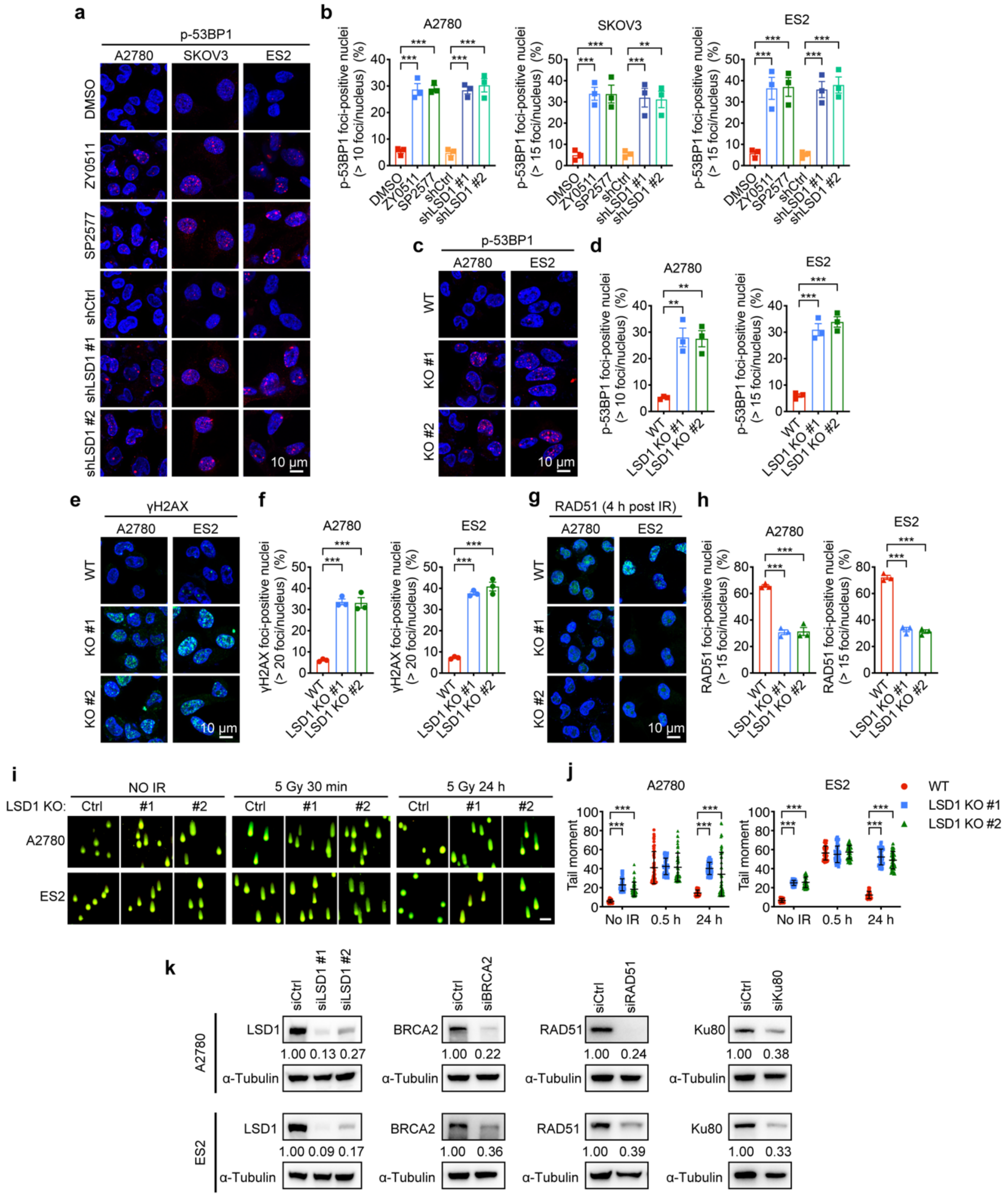

Supplementary Fig. 6 | LSD1 inhibition suppresses HR.

a, **b**, Representative images (**a**) and quantification (**b**) of p-53BP1-foci staining performed in A2780, SKOV3 and ES2 cells with or without 1 µM LSD1i (ZY0511 or SP2577) for 48 h or LSD1 knockdown (shLSD1) treatment. Red, p-53BP1; blue, DAPI. Scale bar, 10 µm. Data represent mean ± SEM of three biologically independent experiments (unpaired two-tailed Student's *t* test).

c, **d**, Representative images (**c**) and quantification (**d**) of p-53BP1-foci staining performed in

A2780 and ES2 cells before and after LSD1 knockout. Red, p-53BP1; blue, DAPI. Scale bar, 10 μm. Data represent mean ± SEM of three biologically independent experiments (unpaired two-tailed Student's *t* test).

e, **f**, Representative images (**e**) and quantification (**f**) of γH2AX-foci staining performed in A2780 and ES2 cells before and after LSD1 knockout. Green, γH2AX; blue, DAPI. Scale bar, 10 μm. Data represent mean ± SEM of three biologically independent experiments (unpaired two-tailed Student's *t* test).

g, **h**, Representative images (**g**) and quantification (**h**) and of RAD51 nuclear foci in A2780 and ES2 cells before and after LSD1 knockout at 4 h after 2 Gy IR treatment. Green, RAD51; blue, DAPI. Scale bar, 10 μm. Data represent mean ± SEM of three biologically independent experiments (unpaired two-tailed Student's *t* test).

i, **j**, Representative images (**i**) and quantification (**j**) of neutral comet assays in A2780 and ES2 cells before and after LSD1 knockout at the indicated time after 5 Gy IR treatment. Scale bar, 100 μm. DNA damage was quantified via the tail moment using the CometScore software. Data represent mean ± SEM (unpaired two-tailed Student's *t* test). The experiments were repeated three times.

k, Western blot analysis of indicated proteins in A2780 DR-GFP, ES2 DR-GFP, A2780 EJ5-GFP and ES2 EJ5-GFP cells transfected with indicated siRNAs and control siRNAs.

ns, not significant, $p > 0.05$; *$p < 0.05$; **$p < 0.01$; ***$p < 0.001$. Source data and exact *p* values are provided as the Source Data file.

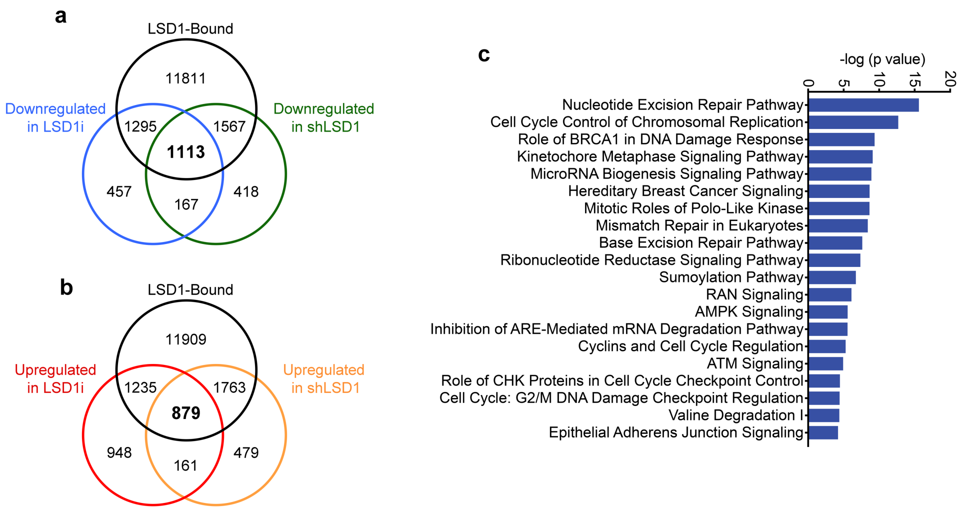

Supplementary Fig. 7 | CUT&Tag-seq and RNA-seq analysis of LSD1 inhibition on DNA repair genes expression.

a, b, Venn diagram showing the overlap of the up- or down-regulated genes in RNA-seq after LSD1 knockdown or LSD1i (ZY0511)-treated with the genes bound by LSD1 in CUT&Tag-seq in ES2 cells.

c, Ingenuity Pathway Analysis of the top twenty significantly regulated pathways of the 1113 overlap genes showed in (**a**) that were both downregulated by LSD1 inhibition and directly bound by LSD1.

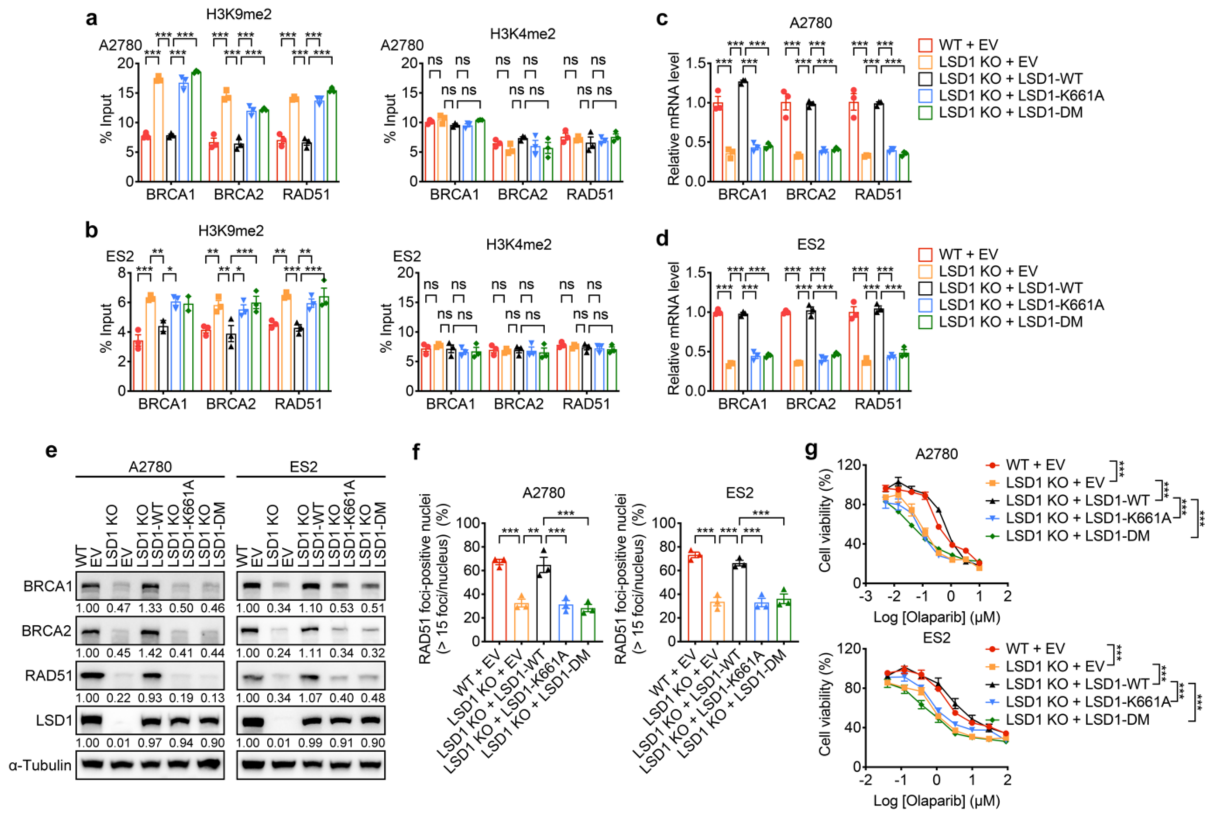

Supplementary Fig. 8 | Enzymatic activity of LSD1 is essential for H3K9me2 demethylation at BRCA1/2 and RAD51 gene loci.

a, **b**, ChIP-qPCR analysis showing the enrichment levels of H3K9me2 and H3K4me2 at the BRCA1, BRCA2 and RAD51 gene promoter in LSD1 knockout A2780 (**a**) and ES2 (**b**) cells overexpressing wild-type LSD1 (LSD1-WT), catalytically deficient mutant LSD1 (LSD1-K661A and LSD1-DM), or empty vector (EV). Data represent the percent of total chromatin input ± SEM of three biologically independent experiments (unpaired two-tailed Student's *t* test; ns, not significant).

c, **d** RT-qPCR analysis of indicated gene expression in LSD1 knockout A2780 (**c**) and ES2 (**d**) cells overexpressing wild-type LSD1 (LSD1-WT), catalytically deficient mutant LSD1 (LSD1-K661A and LSD1-DM), or empty vector (EV). GAPDH was used as the loading control. Data represent mean ± SEM of three biologically independent experiments (unpaired two-tailed Student's *t* test).

e, Western blot analysis of indicated proteins in LSD1 knockout A2780 and ES2 cells overexpressing wild-type LSD1 (LSD1-WT), catalytically deficient mutant LSD1 (LSD1-K661A and LSD1-DM), or empty vector (EV). α-Tubulin was used as the loading control. Numbers below western blot panels represent relative quantification of the respective

bands normalized to loading control by densitometry.

f, Quantification of RAD51 nuclear foci at 4 h after 2 Gy IR treatment in LSD1 knockout A2780 and ES2 cells overexpressing wild-type LSD1 (LSD1-WT), catalytically deficient mutant LSD1 (LSD1-K661A and LSD1-DM), or empty vector (EV). At least 100 cells were analyzed for each condition. Data represent mean ± SEM of three biologically independent experiments (unpaired two-tailed Student's *t* test).

g, Cell viability in response to olaparib in LSD1 knockout A2780 and ES2 cells overexpressing wild-type LSD1 (LSD1-WT), catalytically deficient mutant LSD1 (LSD1-K661A and LSD1-DM), or empty vector (EV). Data represent mean ± SEM of three biologically independent experiments (two-way ANOVA).

ns, not significant, $p > 0.05$; *$p < 0.05$; **$p < 0.01$; ***$p < 0.001$. Source data and exact *p* values are provided as the Source Data file.

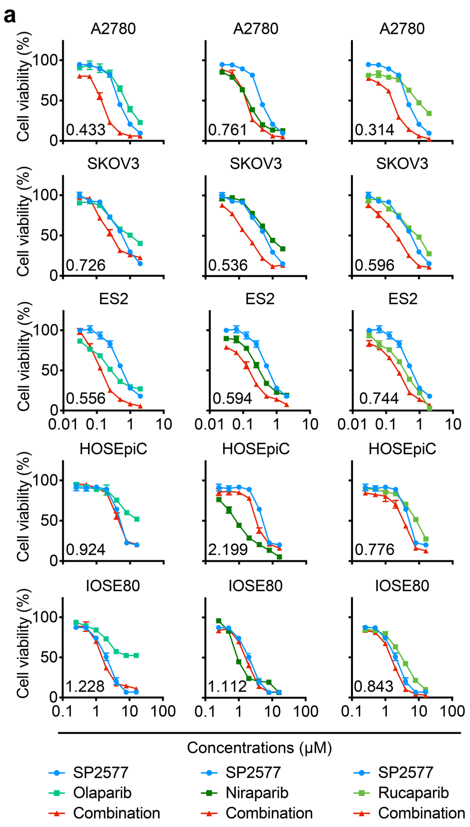

Supplementary Fig. 9 | Effect of PARPi and LSD1i on proliferation of different cell lines.

a, Dose-response curves for LSD1i (SP2577) or PARPi (olaparib, niraparib, or rucaparib) alone or combined for 72 h in A2780, SKOV3 and ES2 cell lines. Combination index (CI) was calculated using CompuSyn software with the Chou-Talalay equation. Data represent mean ± SEM of three biologically independent experiments. Source data are provided as the Source Data file.

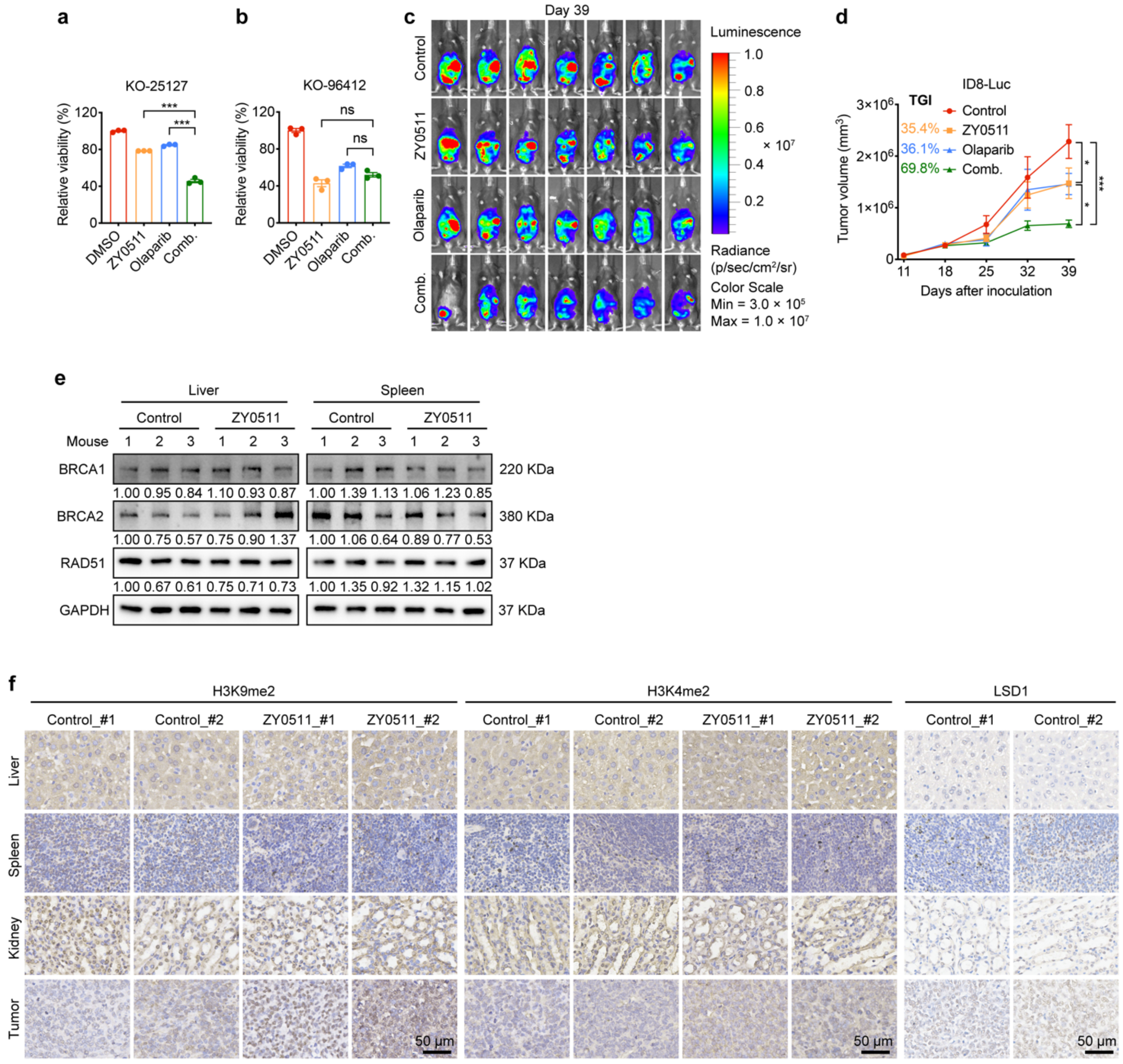

Supplementary Fig. 10 | Effect of olaparib and LSD1i *in vivo*.

a, **b** Cell viability of PDOs treated with vehicle, LSD1i, olaparib alone or their combination for 5 days. Data represent mean ± SEM normalized to vehicle cells (n = 3 biologically independent experiments).

c, **d** Representative living luminescence images (**c**) and quantification of the luciferase fluorescence signal intensity (**d**) of ID8-Luc syngeneic model mice treated with vehicle (30 mg/mL PEG4000 plus 12 mg/mL Tween 20 in water and 5% DMSO plus 30% PEG300 in water intraperitoneally 5 days on, 2 days off schedule), ZY0511 (intraperitoneally 30 mg/kg 5 days on, 2 days off schedule), olaparib (intraperitoneally 30 mg/kg 5 days on, 2 days off schedule), or a combination of ZY0511 and olaparib. Data represent mean ± SEM

(n = 7 mice per group; two-way ANOVA).

e, Western blot analysis of indicated proteins in selected tissues from control and ZY0511-treated mice. GAPDH was used as loading control. Numbers below western blot panels represent relative quantification of the respective bands normalized to loading control by densitometry.

f, IHC of the indicated proteins in selected tissues from control and ZY0511-treated mice. Scale bar, 50 μm.

ns, not significant, $p > 0.05$; $^{*}p < 0.05$; $^{**}p < 0.01$; $^{***}p < 0.001$. Source data and exact *p* values are provided as the Source Data file.

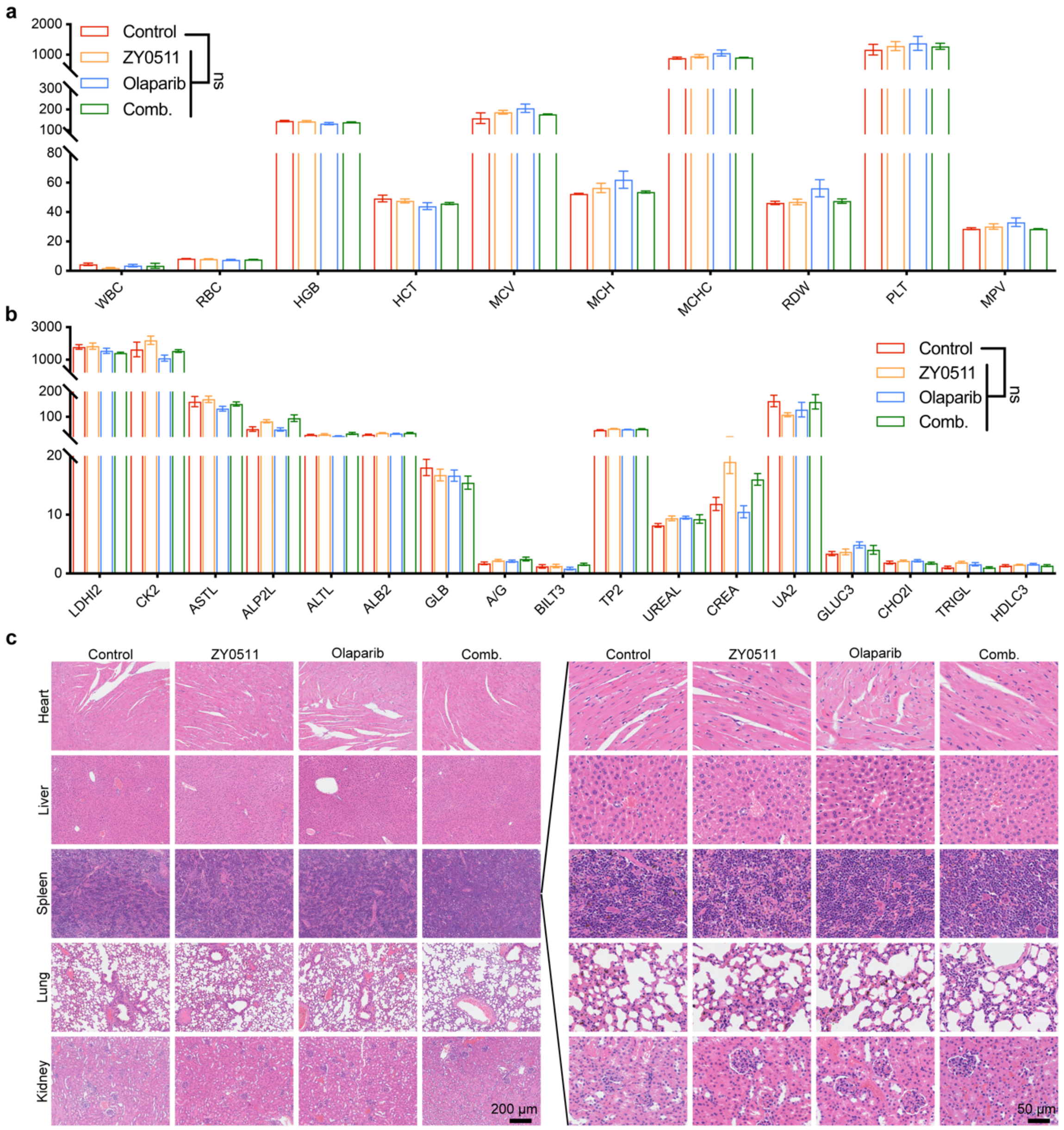

Supplementary Fig. 11 | Safety profile of a combination of olaparib and LSD1i *in vivo*.

a, Blood routine assay performed at the end of treatment. Data represent mean ± SEM (n = 6 mice per group; unpaired two-tailed Student's *t* test).

b, Blood biochemical assay performed at the end of treatment. Data represent mean ± SEM (n = 6 mice per group; unpaired two-tailed Student's *t* test).

c, Representative H&E staining images of the heart, liver, spleen, lung, and kidney at the end of the dosing.

ns, not significant, $p > 0.05$; *$p < 0.05$; **$p < 0.01$; ***$p < 0.001$. Source data and exact *p* values are provided as the Source data file.

Supplementary Table 1. Clinicopathologic characters of OC patients.

Characteristic	Number (n)	%
**Patients**	45	/
**Gender**		
Female	45	100%
**Age (years)**	33-72	/
**Subtypes**		
Serous carcinoma	36	80%
Mucinous carcinoma	0	0
Endometrioid carcinoma	8	17.8%
Clear cell carcinoma	1	2.2%
**TNM stage**		
I	7	15.6%
II	9	20.0%
III	23	51.1%
IV	6	13.3%
**Lymph node involvement**		
Yes	14	31.1%
No	31	68.9%

Supplementary Table 2. Primer sequences of RT-qPCR and ChIP-qPCR.

RT-qPCR primer sequences:

Gene Symbol	Forward Primer Sequence 5'-3'	Reverse Primer Sequence 5'-3'
ATM	GCCGTGATGACCTGAGACAAGATG	CAAGAACACCACTTCGCTGAGAGAG
ATR	CACCACCAGACAGCCTACAATGC	CCAGAGCCACTTTGCCCTTTCC
CHEK1	CCTTTGTGGAAGACTGGGACTTGG	TCTACGGCACGCTTCATATCTACAATC
CHEK2	CCAGCCAGTCCTCTCACTCCAG	GTTCTTGGTCCTCAGGTTCTTGGTC
CDKN1A	GCTGAGCCGCGACTGTGATG	CCTCCAGTGGTGTCTCGGTGAC
RNF8	CCTGATGATTTCTCGAAACCAC	TGTTCAGCCAAACACCATTTAG
RNF168	CATACAGAGGTTGTTGGCAGAGGAG	TCAGTTGTTCTTCCATCGCTCTTCG
MRE11	TCTGCTTCTGCCTTTAGTGCTGATG	TGTTGGTTGCTGCTGAGATGCTATC
RAD50	AGCCGAAAGAAGCAAATGGAGAAAG	TTCCAGTTCACGATGACAGTCTACC
NBS1	AGCAGCAGACCAACTCCATCAG	GGGTGTAGCAGGTTGTGTTTGTTC
RBBP8	AAGCAGCAGATGAAGAGGAGGAATTG	TACGGCTCCACAAACGCTTTCTG
EXO1	ATGACTACAATCCAGACACTGCTATGC	TGGGAGAGTAATTCCTATGCCAAATGC
RPA1	CGGGAATGGGTTCTACTGTTTCTAAGG	GTAAGGAGTGAGGCTGGCAATGG
RPA2	CTTTGGTGGGAATAGCTTCATG	GCTTGCTTGATTGAGGATACAG
BRCA1	GAACGGGCTTGGAAGAAAAT	GTTTCACTCTCACACCCAGA
BRCA2	AGTCAGTGGTATGTGGGAGTTTGTTTC	TAGAACTAAGGGTGGGTGGTGTAGC
PALB2	TCTGGCACATGCACAGGACA	TATCTTCAGTGGGCCCAGCG
RAD51	GCCCTTTACAGAACAGACTACT	TTGAGCTACCACCTGATTAGTG
ATRX	AATGTAGGTGGTGTGCGGAAGG	CAAAGGCTCTGGGTGACAAATGTAG
TP53BP1	CATCGTCACATGAGAACAATCC	TTTCACACTCCTGACACTCTAC
XRCC6	ACTGCTGAAGAAACACCATTACCTGAG	CAACCTCCTTCTCCAGACACTTGATG
XRCC5	GGAAGCCTCTGGAAGTTCTGTCAC	AATCGTCCACATCACCACCTTCTTC
PRKDC	CAGAGAACATGGCAGGAGAGAATCAG	TGAAGACACAGCAGATGACAGATATGG
DCLRE1C	AGGAGTCCAGGTTCATGTGAATAAGC	ATCTGAGTGTTGCGGTCTGTTGTG
NHEJ1	AACAGGTGTGGCATGAACAGGTG	TGAGCAGCGTCCTTCAACAATGG
LIG4	CAACCTCTATCCATCTACAAGCCAGAC	CATTCCACCCCGTGATCCTTTACC

ChIP-qPCR primer sequences:

Gene Symbol	Forward Primer Sequence 5'-3'	Reverse Primer Sequence 5'-3'
BRCA1	TACCCAGAGCAGAGGGTGAA	ACGGAAAAGCGCGGGAATTA
BRCA2	CGAGCTTCTGAAACTAGGCG	AATCTGTCCCCTCACGCTTC
RAD51	GAGAAGTGGAGCGTAAGCCA	CACACACTCACCTCGGTCC
