# Supplementary material for: Repression of LSD1 potentiates homologous recombination-proficient ovarian cancer to PARP inhibitors through down-regulation of BRCA1/2 and RAD51
Source: Nat Commun. 2023 Nov 16;14:7430. doi: 10.1038/s41467-023-42850-x (PMC10654398; doi:10.1038/s41467-023-42850-x)
Supplement: Supplementary file 3 — Reporting Summary [file 41467_2023_42850_MOESM3_ESM.pdf]

Corresponding author(s): Yinglan Zhao

Last updated by author(s): Oct 11, 2023

Reporting Summary

Nature Portfolio wishes to improve the reproducibility of the work that we publish. This form provides structure for consistency and transparency in reporting. For further information on Nature Portfolio policies, see our Editorial Policies and the Editorial Policy Checklist.

Statistics

For all statistical analyses, confirm that the following items are present in the figure legend, table legend, main text, or Methods section.

n/a	Confirmed	
☐	☒	The exact sample size (*n*) for each experimental group/condition, given as a discrete number and unit of measurement
☐	☒	A statement on whether measurements were taken from distinct samples or whether the same sample was measured repeatedly
☐	☒	The statistical test(s) used AND whether they are one- or two-sided<br>*Only common tests should be described solely by name; describe more complex techniques in the Methods section.*
☒	☐	A description of all covariates tested
☐	☒	A description of any assumptions or corrections, such as tests of normality and adjustment for multiple comparisons
☐	☒	A full description of the statistical parameters including central tendency (e.g. means) or other basic estimates (e.g. regression coefficient) AND variation (e.g. standard deviation) or associated estimates of uncertainty (e.g. confidence intervals)
☐	☒	For null hypothesis testing, the test statistic (e.g. *F*, *t*, *r*) with confidence intervals, effect sizes, degrees of freedom and *P* value noted<br>*Give P values as exact values whenever suitable.*
☒	☐	For Bayesian analysis, information on the choice of priors and Markov chain Monte Carlo settings
☒	☐	For hierarchical and complex designs, identification of the appropriate level for tests and full reporting of outcomes
☐	☒	Estimates of effect sizes (e.g. Cohen's *d*, Pearson's *r*), indicating how they were calculated

Our web collection on statistics for biologists contains articles on many of the points above.

Software and code

Policy information about availability of computer code

Data collection	All software is commercially or freely available. Images were captured using a fluorescence microscope (Olympus CKX53) or Lecia TCS SP8 laser scanning microscope. Flow cytometry analysis was performed on a NovoCyte Advanteon cytometer (ACEA Biosciences, CA). For IHC analysis, slides were photographed by Vectra Polaris (Perkin Elmer). For RNA-seq, CUT&Tag and ATAC-seq, the Illumina NovaSeq 6000 was used. For bioluminescent imaging, mice were imaged on PerkinElmer IVIS Lumina III (PerkinElmer).
Data analysis	All software is commercially or freely available. Synergistic effects between both compounds were calculated using the Chou-Talalay equation in CompuSyn software (http://www.combosyn.com). Flow cytometry analysis was performed on NovoExpress 1.6.1 (Agilent Biosciences). Neutral comet assays were analyzed with the CometScore software. The Radiance (photons) within each area of interest was determined using the Living Image Software 4.5.2 (Perkin Elmer). For CUT&Tag-seq and ATAC-seq analysis, the bowtie2 (v2.3.4.2, RRID:SCR_016368) was used to align the sequencing data with the hg19 as the reference with parameters of --very-sensitive -X 2000 and the duplicated reads were filter by the GATK pipeline (v4.1.3.0, RRID:SCR_001876). The DESeq2 (v1.26.0, RRID:SCR_015687) pipeline was used to identify the significantly differential regions with cutoff of p-value < 0.05 and |log2 fold change| > 0.5. The ChIPseeker (v1.22.1, RRID:SCR_021322) was used to annotate the peaks signal and regions in each sample. GSEA was performed using GSEA software (http://www.gsea-msigdb.org/gsea/) with 1000 permutations. Gene sets used were obtained from MSigDB (Hallmark gene sets; reactome subset of canonical pathway from C2 databases). Statistical Analysis was done in GraphPad Prism 9. Figures were compiled using Fiji (v2.0.0-rc-69/1.52p)and Adobe Photoshop 2022. Schematic diagrams in Fig. 8a, h are created with BioRender.com (https://biorender.com/).

For manuscripts utilizing custom algorithms or software that are central to the research but not yet described in published literature, software must be made available to editors and reviewers. We strongly encourage code deposition in a community repository (e.g. GitHub). See the Nature Portfolio guidelines for submitting code & software for further information.

Data

Policy information about availability of data

All manuscripts must include a data availability statement. This statement should provide the following information, where applicable:

- Accession codes, unique identifiers, or web links for publicly available datasets
- A description of any restrictions on data availability
- For clinical datasets or third party data, please ensure that the statement adheres to our policy

The RNA-seq, CUT&Tag-seq and ATAC-seq data were submitted to the Gene Expression Omnibus (GEO) database under accession number: GSE218798 (https://www.ncbi.nlm.nih.gov/geo/query/acc.cgi?acc=GSE218798). TCGA and Cancer Cell Line Encyclopedia (CCLE) dataset of OC was downloaded from cBioPortal (https://www.cbioportal.org/results/download?cancer_study_list=ov_tcga_pub&Z_SCORE_THRESHOLD=2.0&RPPA_SCORE_THRESHOLD=2.0&profileFilter=mutations%2Cgistic&case_set_id=ov_tcga_pub_cna_seq&gene_list=KDM1A&geneset_list=%20&tab_index=tab_visualize&Action=Submit; https://www.cbioportal.org/results/download?cancer_study_list=ccle_broad_2019&Z_SCORE_THRESHOLD=2.0&RPPA_SCORE_THRESHOLD=2.0&profileFilter=mutations%2Cstructural_variants%2Ccna&case_set_id=ccle_broad_2019_cnaseq&gene_list=KDM1A&geneset_list=%20&tab_index=tab_visualize&Action=Submit). Other OC datasets were downloaded from the Oncomine (https://www.oncomine.org/resource/; The service has been terminated on January 17, 2022.), Ualcan (https://ualcan.path.uab.edu/cgi-bin/CPTAC-Result.pl?genenam=KDM1A&ctype=OV), muTarget (https://www.mutarget.com/) , Kaplan-Meier Plotter (https://kmplot.com/analysis/index.php?p=service&cancer=ovar)and the ROC Plotter (https://www.rocplot.org/ovarian/index) database.hg19 was downloaded from UCSC (https://hgdownload.soe.ucsc.edu/goldenPath/hs1/vsHg19/). Source data are provided with this paper.

Human research participants

Policy information about studies involving human research participants and Sex and Gender in Research.

Reporting on sex and gender	Sex or gender was not considered in the study design. However, as most ovarian cancer patients were female, our ovarian biospecimens were from female patients.
Population characteristics	The patients ranged from 26 to 70 years old. The detailed patient characteristics in TMA were listed in Supplementary Table 1.
Recruitment	We obtained paraffin-embedded FTE, HOSE, and OC tissues of patients from the West China Hospital, Sichuan University. Notably, there was no evident self-selection bias identified in this study.
Ethics oversight	The paraffin-embedded FTE, HOSE, and OC tissues of patients were collected with the approval of the Biomedical Ethics Review Committee, West China Hospital, Sichuan University (permit number: 2018SZ0241).

Note that full information on the approval of the study protocol must also be provided in the manuscript.

Field-specific reporting

Please select the one below that is the best fit for your research. If you are not sure, read the appropriate sections before making your selection.

☒ Life sciences ☐ Behavioural & social sciences ☐ Ecological, evolutionary & environmental sciences

For a reference copy of the document with all sections, see nature.com/documents/nr-reporting-summary-flat.pdf

Life sciences study design

All studies must disclose on these points even when the disclosure is negative.

Sample size	For in vitro assays, n = 3 biologically independent experiments were used for sample size,following standard practices in the research field. This selection was made to produce reproducible results with a significance level of less than 0.05 and a power exceeding 90%.<br>For the in vivo mouse studies, we determined the sample size based on our extensive experience with animal models and endpoints. mice were randomized into treatment and control groups (n ≥ 6 mice per group), as described in the manuscript, to ensure the generation of reproducible results with a significance level of less than 0.05 and a power of over 90%.<br>Regarding human samples, the total sample size was contingent upon the availability of samples and the characteristics of the patients.
Data exclusions	No data were excluded from analyses.
Replication	Replicates were set up in all cell line experiments and animal experiments as showed in text, figure legends. All other experiments have been repeated at least twice with consonant results.
Randomization	Mice were randomized prior to any treatment. All the cell experiments in vitro were performed by plating the cells in independent dishes or plates, and randomly assigned to experimental or control groups and no bias was introduced. Detailed definitions and descriptions were provided in the manuscript.

Blinding	In vitro assays were not conducted in a blinded manner as the same investigator both planned and performed the experiment. Investigators were blinded to group allocation during data collection and analysis wherever possible. Xenograft tumors were randomly assigned into treatment or control groups. Tumor growth measurements were performed blindly.

Reporting for specific materials, systems and methods

We require information from authors about some types of materials, experimental systems and methods used in many studies. Here, indicate whether each material, system or method listed is relevant to your study. If you are not sure if a list item applies to your research, read the appropriate section before selecting a response.

Materials & experimental systems

n/a	Involved in the study
☐	☒ Antibodies
☐	☒ Eukaryotic cell lines
☒	☐ Palaeontology and archaeology
☐	☒ Animals and other organisms
☒	☐ Clinical data
☒	☐ Dual use research of concern

Methods

n/a	Involved in the study
☐	☒ ChIP-seq
☐	☒ Flow cytometry
☒	☐ MRI-based neuroimaging

Antibodies

Antibodies used	The primary antibodies for western blot analysis were diluted as followed: rabbit anti-LSD1 (Cell Signaling Technology, 2139S and 2184S, 1:1000), rabbit anti-H3 (Abcam, ab1791, 1:5000), rabbit anti-H3K4me1 (Abcam, ab8895, 1:1000), rabbit anti-H3K4me2 (Abcam, ab32356, 1:1000), mouse anti-H3K9me1 (Abcam, ab8896, 1:1000), mouse anti-H3K9me2 (Abcam, ab1220, 1:1000), rabbit anti-H3K9me3 (Cell Signaling Technology, 13969S, 1:1000), mouse anti-γH2AX (Millipore, 05-636, 1:1000), rabbit anti-ATM (Cell Signaling Technology, 2873T, 1:1000), rabbit anti-phospho-ATM (Ser1981) (Cell Signaling Technology, 13050T, 1:1000), rabbit anti-RAD51 (Cell Signaling Technology, 8875S, 1:1000), rabbit anti-BRCA1 (Cell Signaling Technology, 9010S, 1:1000; Proteintech, 22362-1-AP, 1:500), rabbit anti-BRCA2 (Cell Signaling Technology, 10741S, 1:1000; ABclonal, A2435, 1:500), rabbit anti-RAD54 (Cell Signaling Technology, 15016T, 1:1000), rabbit anti-p95/NBS1 (Cell Signaling Technology, 14956T, 1:1000), rabbit anti-CtIP (Cell Signaling Technology, 9201S, 1:1000), rabbit anti-phospho-Chk2 (Thr68) (Cell Signaling Technology, 2197T, 1:1000), rabbit anti-phospho-p53 (Ser15) (Cell Signaling Technology, 9286S, 1:1000), rabbit anti-phospho-ATR (Ser428) (Abcam, ab178407, 1:1000), rabbit anti-ATR (Proteintech, 19787-1-AP, 1:1000), rabbit anti-DNAPKcs (Cell Signaling Technology, 38168T, 1:1000), rabbit anti-phospho-DNAPKcs (Ser2056) (Cell Signaling Technology, 68716T, 1:1000), rabbit anti-Ku70 (Cell Signaling Technology, 4588T, 1:1000), rabbit anti-Ku80 (Cell Signaling Technology, 2180T, 1:1000), rabbit anti-DNA Ligase IV (Cell Signaling Technology, 14649T, 1:1000), rabbit anti-XLF (Cell Signaling Technology, 2854T, 1:1000), rabbit anti-Artemis (Cell Signaling Technology, 13381T, 1:1000), rabbit anti-53BP1 (Abcam, ab175933, 1:1000), rabbit anti-phospho-53BP1 (Ser1778) (Cell Signaling Technology, 2675S, 1:1000), rabbit anti-RPA32/RPA2 (Abcam, ab76420, 1:1000), rabbit anti-RPA70 (Abcam, ab79398, 1:1000), rabbit anti-RAD50 (Abcam, ab124682, 1:1000), rabbit anti-MRE11 (Abcam, ab208020, 1:1000), rabbit anti-phospho-RPA32 (Ser4/Ser8) (Bethyl Laboratories, A300-245A, 1:1000), rabbit anti-phospho-RPA32 (Ser33) (Bethyl Laboratories, A300-246A, 1:1000), rabbit anti-Caspase 3 (Cell Signaling Technology, 14220T, 1:1000), rabbit anti-Cleaved Caspase 3 (Asp175) (Cell Signaling Technology, 9664T, 1:1000), mouse anti-Caspase 9 (Cell Signaling Technology, 9508T, 1:1000), rabbit anti-Cleaved Caspase 9 (Asp330) (Cell Signaling Technology, 52873T, 1:1000), rabbit anti-PARP (Cell Signaling Technology, 9542T, 1:1000), rabbit anti-Cleaved PARP (Asp214) (Cell Signaling Technology, 5625T, 1:1000), rabbit anti-α-Tubulin (Proteintech, 66031-1-Ig, 1:2000), rabbit anti-GAPDH (Cell Signaling Technology, 8884S, 1:2000), rabbit anti-β-actin (Cell Signaling Technology, 12620S, 1:2000).<br>The following antibodies were used to perform immunofluorescence: mouse anti-γH2AX (Millipore, 05-636, 1:500), rabbit anti-RAD51 (Abcam, ab133534, 1:250), rabbit anti-phospho-53BP1 (Ser1778) (Cell Signaling Technology, 2675S, 1:500).<br>The following antibodies were used to perform CUT & Tag and ChIP: anti-H3K4me2 (Abcam, ab32356, 4 ug for each Ig), anti-H3K9me2 (Abcam, ab1220, 4 ug for each Ig), anti-LSD1 (Millipore, 17-10531, 2 ug for each Ig).<br>The following antibodies were used to perform Immunohistochemistry: rabbit anti-Cleaved Caspase 3 (Asp175) (Cell Signaling Technology, 9664S, 1:50), rabbit anti-LSD1 (Cell Signaling Technology, 2139S, 1:100), rabbit anti-Ki67 (Abcam, ab1667, 1:100), rabbit anti-γH2AX (Cell Signaling Technology, 9718S, 1:100); rabbit anti-H3K4me2 (Cell Signaling Technology, 9725S, 1:100), rabbit anti-H3K9me2 (ABclonal, A2359, 1:50).
Validation	All antibodies used in this study are commercially available, and were otherwise validated by the manufacturer, by previous studies from other laboratories or by previous studies from our laboratory, as cited in the text and methods. For example, we used CRISPR-Cas9 to knockout the target proteins and validated knockout by western blot.

Eukaryotic cell lines

Policy information about cell lines and Sex and Gender in Research

Cell line source(s)	The human OC cell lines A2780 (Cat. # 93112519) and COV362 (Cat. # 07071910) were purchased from Sigma. The human OC cell lines SKOV3 (Cat. # HTB-77), ES2 (Cat. # CRL-1978), and OVCAR3 (Cat. # HTB-161) were purchased from American Type Culture Collection (ATCC). The human OC cells Kuramochi (Cat. # JCRB0098) were purchased from Japanese Collection of Research Bioresources (JCRB) Cell Bank. The human ovarian epithelial cells HOSEpiC (Cat. #7310) were purchased from ScienCell Research Laboratories. The nonmalignant human ovarian surface epithelial cells IOSE80 (Cat. # CTCC-400-0117)

	were purchased from Meisen Chinese Tissue Culture Collection. The mouse OC cells ID8 were kindly gifted by Dr. Zhou at Sichuan University. The A2780, OVCAR3, IOSE80 and HOSEpiC were cultured in RPMI 1640 medium (Gibco) with 10% fetal bovine serum (FBS). The SKOV3, ES2, COV362, Kuramochi and ID8 cells were cultured in Dulbecco's modified Eagle's medium (DMEM) (Gibco) with 10% FBS. UWB1.289 cells were kindly gifted by Dr. Yu at Westlake University and were cultured RPMI 1640 : MEGM (1 : 1) with 3% FBS (Lonza, CC-3150). All cell lines were maintained in standard conditions at 37 °C and 5% CO2.
Authentication	All cell lines were authenticated by fingerprinting using short tandem repeat testing.
Mycoplasma contamination	All cell lines were verified to be free of mycoplasma contamination.
Commonly misidentified lines (See ICLAC register)	No commonly misidentified cell lines were used in this study.

Animals and other research organisms

Policy information about studies involving animals; ARRIVE guidelines recommended for reporting animal research, and Sex and Gender in Research

Laboratory animals	Five- to six-week-old female BALB/c nude mice and C57BL/6 mice were purchased from Charles River Laboratories (Beijing, China). Five- to six-week-old female immunodeficient NCG mice were purchased from GemPharmatech (Nanjing, China). The mice were housed in specific pathogen-free conditions with controlled temperature (22-26°C), humidity (55±5%), and a 12h light/dark cycle, with 5 mice per cage.
Wild animals	No wild animals were used in the study.
Reporting on sex	Sex was not considered in the study design. Only female mice were used in this study, due to our study is focused on ovarian cancer which overwhelmingly affects female subjects.
Field-collected samples	No field-collected samples were used in this study.
Ethics oversight	The animal experiments were performed in strict accordance with the People's Republic of China Legislation Regarding the Use and Care of Laboratory Animals. All protocols used in this study were approved by the Institutional Animal Care and Treatment Committee of Sichuan University in China (permit number: 20180106).

Note that full information on the approval of the study protocol must also be provided in the manuscript.

ChIP-seq

Data deposition

☒ Confirm that both raw and final processed data have been deposited in a public database such as GEO.

☒ Confirm that you have deposited or provided access to graph files (e.g. BED files) for the called peaks.

Data access links *May remain private before publication.*	GSE218798.
Files in database submission	36 fq.gz files and 18 bw files.
Genome browser session (e.g. UCSC)	IGV_2.13.0

Methodology

Replicates	three replicates
Sequencing depth	We sequenced for ~6G per sample of Illumina NovaSeq 6000 with the mode of paired-end 150bp.
Antibodies	anti-H3K4me2 (Abcam, ab32356, 4 ug for each Ig), anti-H3K9me2 (Abcam, ab1220, 4 ug for each Ig), anti-LSD1 (Millipore, 17-10531, 2 ug for each Ig).
Peak calling parameters	The bowtie2 (v2.3.4.2, RRID:SCR_016368) was used to align the sequencing data with the hg19 as the reference with parameters of --very-sensitive -X 2000. The samtools (v0.1.18, RRID:SCR_002105) was used to convert the sam files to bam files. The MarkDuplicates implemented in GATK (v4.1.3.0, RRID:SCR_001876) was used to filter the duplicated alignment reads in bam files. The SEACR (v1.3, RRID:SCR_001876) was used to call the peak signals and regions in each sample by selecting the top 1% of regions by area under the curve (AUC). The bamCoverage (v3.5.0) was used to generate the bw files in each sample with BPM normalization. The deeptools (v3.5.0, RRID:SCR_016366) was used to visualize the global signal of peaks on TSS regions. The getCounts implemented in chromVAR (v1.8.0) was used to quantify the counts of each peak signal in each sample.
Data quality	For LSD1, a average number of 43208 peaks in each sample were identified.<br>For H3K9me2, a average number of 49590 peaks in each sample were identified.<br>For H3K4me2, a average number of 30168 peaks in each sample were identified.

Software	The Illumina NovaSeq 6000 was used to sequence the library of CUT&Tag-seq assay in each sample with the mode of paired-end 150bp. The bowtie2 (v2.3.4.2, RRID:SCR_016368) was used to align the sequencing data with the hg19 as the reference with parameters of --very-sensitive -X 2000. The samtools (v0.1.18, RRID:SCR_002105) was used to convert the sam files to bam files. The MarkDuplicates implemented in GATK (v4.1.3.0, RRID:SCR_001876) was used to filter the duplicated alignment reads in bam files. The SEACR (v1.3, RRID:SCR_001876) was used to call the peak signals and regions in each sample by selecting the top 1% of regions by area under the curve (AUC). The bamCoverage (v3.5.0) was used to generate the bw files in each sample with BPM normalization. The deeptools (v3.5.0, RRID:SCR_016366) was used to visualize the global signal of peaks on TSS regions. The getCounts implemented in chromVAR (v1.8.0) was used to quantify the counts of each peak signal in each sample. The regions detected less than 10 counts would be filtered for subsequent analysis. The DESeq2 (v1.26.0, RRID:SCR_015687) was used to remove the effects of library size and region length in counts data. The statistic values and variation degree of peaks signal and regions were calculated by DESeq2 (v1.26.0). The significantly differential peaks were selected by the cutoff of p-value < 0.05 and | log2 fold change | > 0.5. The ChIPseeker (v1.22.1, RRID:SCR_021322) was used to annotate the peaks signal and regions in each sample.

Flow Cytometry

Plots

Confirm that:

☒ The axis labels state the marker and fluorochrome used (e.g. CD4-FITC).

☒ The axis scales are clearly visible. Include numbers along axes only for bottom left plot of group (a 'group' is an analysis of identical markers).

☒ All plots are contour plots with outliers or pseudocolor plots.

☒ A numerical value for number of cells or percentage (with statistics) is provided.

Methodology

Sample preparation	Cells was trypsinized and harvested to create cell suspensions in PBS.
Instrument	NovoCyte Advanteon cytometer (ACEA Biosciences, CA)
Software	NovoExpress 1.6.1 (Agilent Biosciences).
Cell population abundance	Cell population data were collected on a debris exclusion gate at the time of acquisition of NovoCyte Advanteon cytometer. At least of 10,000 cell events per sample was collected.
Gating strategy	Cell populations were gated on FSC/SSC for cell selection and debris exclusion. Next, a FSC-H/FSC-A plot was used to exclude the doublets. Live cells were further quantified by FITC or PE to determine positive cell populations.

☒ Tick this box to confirm that a figure exemplifying the gating strategy is provided in the Supplementary Information.
